# Supplementary material for: Patterns of Intron Gain and Loss in Fungi
Source: PLoS Biol. 2004 Nov 30;2(12):e422. doi: 10.1371/journal.pbio.0020422 (PMC532390; doi:10.1371/journal.pbio.0020422)
Supplement: Table S1 — Also available at http://genes.mit.edu/NielsenEtAl/. (4.3 MB ZIP). [file pbio.0020422.st001.zip › NielsenEtAl/html/969.html]

AN0241.1.NCU02133.1.MG02625.1.FG08721.1


```
 CLUSTAL W (1.82) Multiple Sequence Alignments - Introns Inserted


Sequence 1: NCU02133.1	167 aa
Sequence 2: FG08721.1	166 aa
Sequence 3: AN0241.1	178 aa
Sequence 4: MG02625.1	164 aa
Alignment Length: 202 aa
Number Identitical Residues: 87 aa
Alignment Score (without introns) 4119


MG02625.1 	MVK-------------AV1AVLRPDPNATVQVSGHVIFEQESESSPTKVTWDFKGCDANA
NCU02133.1	MHYWLEDSTANLFHIRPV~AVVRGDS----NVKGTVIFEQESESAPTTITYDISGNDPNA
FG08721.1 	MNIKLTHSLA-------V~SVLRGDS----KVSGTVVFEQESESAPTTITWDITGNDPNA
AN0241.1  	MFL--------------V~AVLRGDS----KVSGTVTFEQADENSNTTVSWNITGNDPNA
          	*                * :*:* *.    :*.* * *** .*.: *.:::::.* *.**

MG02625.1 	KRAFHIHTFGDNTNGCTSAGPHL1LFLHVVNPHNKEHGAPEDENRHVGDLGNFDTDGQGN
NCU02133.1	KRGFHIHTFGDNTNGCTSAGPHF1------NPHGTTHGDRTAEVRHVGDLGNIETDAQGN
FG08721.1 	KRGFHIHTFGDNTNGCTSAGPHF1------NPHNKTHGAPSDETRHVGDLGNVETDGQGN
AN0241.1  	ERGFHIHQFGDNTNGCTSAGPHF~------NPFGKTHGAPEDEVRHVGDLGNFKTDAEGN
          	:*.**** **************:       **... **    * ********..**.:**

MG02625.1 	ASGSKEDKFIKLIGPESVVG~RTLVVHAGTDDLGRGGHAESKKTGNAGGRPACG------
NCU02133.1	AKGTVTDNLVKLIGPESVIG0RTVVVHAGTDDLGKGGNEESLKTGNAGPRPACG------
FG08721.1 	AKGSVTDSLIKLIGPHSVIG0RTVVIHAGTDDLGKGDGEESLKTGNAGPRPACG------
AN0241.1  	SKGSKTDKLIKLIGAESVLG0RTLVVHAGTDDLGRGDSEESKKTGNAGARPACGEHSSSR
          	:.*:  *.::****..**:* **:*:********:*.  ** ****** *****. ::: 

MG02625.1 	-------VIGISS------------
NCU02133.1	-------VIGISQ------------
FG08721.1 	-------MCILLYFPMVCH------
AN0241.1  	MGVEWTKLMGINRCHRYCCVNSPIK
          	 . . :.:  :      .  .:. .
```
